# Large vessel vasculitis evaluation by CTA: impact of deep-learning reconstruction and “dark blood” technique

**DOI:** 10.1186/s13244-024-01843-0

**Published:** 2024-10-28

**Authors:** Ning Ding, Xi-Ao Yang, Min Xu, Yun Wang, Zhengyu Jin, Yining Wang, Huadan Xue, Lingyan Kong, Zhiwei Wang, Daming Zhang

**Affiliations:** 1 Radiology Department, State Key Laboratory of Complex Severe and Rare Diseases, Peking Union Medical College Hospital, Chinese Academy of Medical Science and Peking Union Medical College, Beijing, China; 2 Canon Medical System, Beijing, China

**Keywords:** Computed tomography angiography, Deep learning, Dark blood, Large-vessel vasculitis, Image reconstruction

## Abstract

**Objectives:**

To assess the performance of the “dark blood” (DB) technique, deep-learning reconstruction (DLR), and their combination on aortic images for large-vessel vasculitis (LVV) patients.

**Materials and methods:**

Fifty patients diagnosed with LVV scheduled for aortic computed tomography angiography (CTA) were prospectively recruited in a single center. Arterial and delayed-phase images of the aorta were reconstructed using the hybrid iterative reconstruction (HIR) and DLR algorithms. HIR or DLR DB image sets were generated using corresponding arterial and delayed-phase image sets based on a “contrast-enhancement-boost” technique. Quantitative parameters of aortic wall image quality were evaluated.

**Results:**

Compared to the arterial phase image sets, decreased image noise and increased signal-noise-ratio (SNR) and CNR_outer_ (all *p* < 0.05) were obtained for the DB image sets. Compared with delayed-phase image sets, dark-blood image sets combined with the DLR algorithm revealed equivalent noise (*p* > 0.99) and increased SNR (*p* < 0.001), CNR_outer_ (*p* = 0.006), and CNR_inner_ (*p* < 0.001). For overall image quality, the scores of DB image sets were significantly higher than those of delayed-phase image sets (all *p* < 0.001). Image sets obtained using the DLR algorithm received significantly better qualitative scores (all *p* < 0.05) in all three phases. The image quality improvement caused by the DLR algorithm was most prominent for the DB phase image sets.

**Conclusion:**

DB CTA improves image quality and provides better visualization of the aorta for the LVV aorta vessel wall. The DB technique reconstructed by the DLR algorithm achieved the best overall performance compared with the other image sequences.

**Critical relevance statement:**

Deep-learning-based “dark blood” images improve vessel wall image wall quality and boundary visualization.

**Key Points:**

- Dark blood CTA improves image quality and provides better aortic wall visualization.
- Deep-learning CTA presented higher quality and subjective scores compared to HIR.
- Combination of dark blood and deep-learning reconstruction obtained the best overall performance.

**Graphical Abstract:**

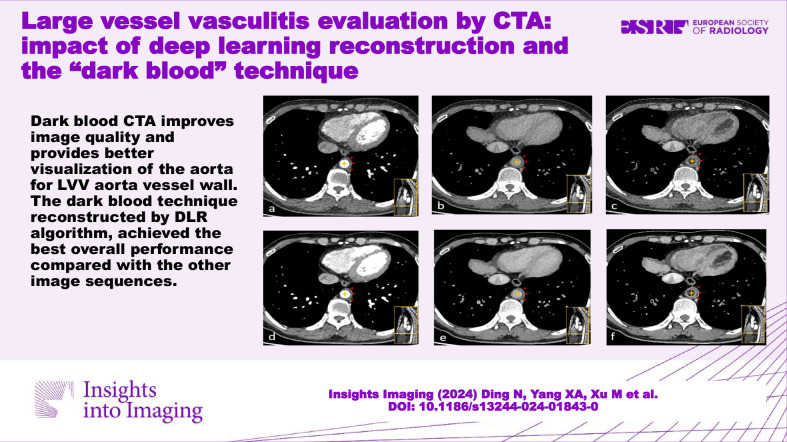

## Introduction

Large-vessel vasculitis (LVV) manifests as inflammation of large blood vessels, such as the aorta and its major branches [1, 2]. Takayasu arteritis (TAK) and giant cell arteritis (GCA) are the most commonly presented forms. An early and accurate diagnosis and treatment can improve patient outcomes [3, 4]. However, the clinical features of LVV are complex and vary with the arteries’ diversity. The initial complaint varies from nonspecific fatigue to inflammatory symptoms such as arthralgia, episcleritis, or cutaneous involvement. Therefore, timely and accurate diagnosis of LVV is still challenging [5, 6].

Multiple noninvasive imaging approaches are available for diagnosis. Magnetic resonance imaging (MRI) and CT are two representative clinical modalities with advantages and disadvantages. Computed tomography angiography (CTA) allows an accurate assessment of stenosis or aneurysm of the aorta [2, 7, 8] and can also demonstrate wall thickening of the entire arterial tree [3, 5]. Therefore, it plays a vital role in the diagnostic workup of LVV because it allows the evaluation of vascular wall inflammation with excellent spatial resolution and shorter procedural time [9–11]. However, in several studies, MRI has shown a higher ability to identify vessel wall edema and inflammation than CTA [12–15]. MRI with the dark blood (DB) technique is beneficial for depicting the surrounding vessel wall with high conspicuity to quantify the vascular wall inflammation appropriately.

Nevertheless, inspired by the MR black blood technique, the visualization capability of CTA of the aortic wall can be strengthened using similar approaches. “Dark Blood” CT angiography images can be obtained through various post-processing approaches, including dual-energy CT material decomposition [16] and preset visualization of DB cinematic rendering [17]. In addition, a new dark-blood CTA algorithm was developed based on a technique called contrast-enhancement boost (CE-boost), previously used to improve the visualization of type II endoleaks after endovascular aortic aneurysm repair [18]. This method comprises two steps based on an accurate deformable registration algorithm. First, arterial-phase CT images are subtracted from delayed-phase CT images, resulting in a suppressed intraluminal signal and enhanced vasculature, and the subtracted images are added to the initial delayed phase with an automatic denoising procedure. Thus, CT images obtained are analogous to DB MRI images.

In addition to the DB CTA method, deep-learning reconstruction (DLR) for CT images has been developed to improve image quality with reduced image noise and improved vessel visualization. DLR algorithm (Advanced Intelligent Clear-IQ Engine (AiCE, Canon Medical System) introduces deep convolutional neural networks trained on high-dose model-based iterative reconstruction CT images into the image reconstruction process, thus producing high-quality images without noise contamination [19]. Studies have shown that the DLR algorithm can improve the image quality of the aorta, portal vein, and liver in various phases of abdominal CT images [20, 21].

We hypothesized that applying the DB CTA technique combined with the DLR algorithm could achieve optimal image quality in aortic CTA examinations, especially for the visualization and quantification of vessel wall inflammation.

This study aimed to assess the impact of DB CTA, DLR, and a combination of the DB CTA and DLR techniques on aortic CTA in terms of image quality and vessel wall visualization for LVV evaluation.

## Materials and methods

### Study population

This research was prospectively performed in a single medical center. Written informed consent was obtained from all the patients. The prospective study protocol was approved by the Institutional Ethics Committee (No. HS-2427) On June 23, 2020.

Sample size estimates were calculated using PASS version 15.0 (Kaysville, Utah, USA), where the module of “one-way repeated measures” was carried out on a pilot study of 10 patients. The prestudy indicated that only a dozen patients were needed to satisfy the power of 90% with a Wilks Lambda Test at a 0.05 significance level.

From June 2020 to October 2022, consecutive adult patients who were clinically diagnosed with LVV (either TAK or GCA) and referred for imaging assessment of the aorta and its branches were enrolled in our study. The exclusion criteria were those aged < 18 years, with contrast-related allergy, impaired renal function, pregnancy, thyrotoxicosis, and inadequate imaging quality. Seventy-two consecutive adult patients were initially enrolled in our study, and 50 were finally included. The mean age of the included 50 patients (43 women, 86%) was 33.5 ± 11.4 (range 18–60) years.

### CTA acquisition

All aortic CTA were performed using a 320-row-detector CT scanner (Aquilion ONE GENESIS Edition; Canon Medical Systems Corp., Otawara, Japan). The scanning sequences included non-contrast, arterial, and delayed phases, and the parameters were as follows: tube voltage, 100 kVp; rotation time, 0.5 s; tube current adjusted automatically with a noise index of 7.5; collimation, 100× 0.5 mm; field of view, 400 mm. All patients received 50–60 mL of IV iodinated contrast agent (iopamidol injection, 370 mg I/mL, Shanghai Bracco Sine Pharmaceutical Corp.) at a rate of 4 mL/s via the antecubital vein using a dual-syringe power injector (Nemoto-Kyorindo, Tokyo), followed by 30–40 mL of saline at the same injection rate. The images were acquired from 2 cm above the thoracic entrance to 2 cm below the lower margin of the pubic bone.

A bolus tracking technique was applied with a trigger threshold of 180 HU in the descending aorta. Arterial phase imaging was initiated. Delayed-phase imaging was performed for 70 s after contrast medium injection.

### CT image reconstruction

Arterial- and delayed-phase images were reconstructed using Hybrid-IR (Adaptive Iterative Dose Reduction 3D, FC08 kernel, Canon Medical Systems; hereafter, hybrid iterative reconstruction (HIR)) and DLR (AiCE, Body Sharp Kernel, Canon Medical Systems), with a slice thickness of 1.0 mm and an interval of 0.8 mm. The corresponding DB CTA images were generated using CE-Boost software (^SURE^Subtraction, Canon Medical Systems) to obtain the DB-HIR and DB-DLR image datasets.

### Qualitative image quality analysis

Two radiologists (with 9 and 19 years of experience in reviewing CTA studies) independently performed qualitative image analysis using a dedicated workstation (Advantage Workstation 4.7; GE Healthcare). One dedicated slice with maximum wall thickening for each patient was selected from the ascending aorta, aortic arch, descending thoracic aorta, or abdominal aorta. The two radiologists independently reviewed the images and were blinded to the type of image reconstruction algorithm used. The six image datasets evaluated included the arterial phase (A-HIR and A-DLR), delayed phase (D-HIR and D-DLR), and DB (DB-HIR and DB-DLR). The initial window width and level were set to 350 and 50 HU, respectively; both parameters were modifiable. Three subjective parameters were evaluated: –outer-wall delineation, inner-wall delineation, and overall image quality. A 4-point scale evaluated outer-wall delineation and inner-wall delineation: 1 = not identified, < 25% circumscribed margins indistinct density between the outer wall and peri-aortic fat (inner wall and lumen); 2 = poorly identified, 25%–50% circumscribed margins somewhat indistinct; 3 = fairly identified, 50–75% circumscribed margins somewhat distinct; 4 = being well identified, > 75% circumscribed margins distinct. The overall image quality was assessed by scoring the interpretability of the aortic wall using a four-point Likert scale: 1 = non-diagnostic, 2 = moderate visualization, 3 = good visualization, and 4 = excellent visualization [22].

### Vessel wall thickness evaluation

Aortic wall thickness was measured on the same axial slice and workstation used for the qualitative evaluation of image quality. Two reviewers independently measured the maximum aortic wall thickness using A-HIR, A-DLR, D-HIR, D-DLR, DB-HIR, and DB-DLR.

### Quantitative image quality analysis

All CT images were manually segmented using the open-source software ITK-SNAP (http://www.itksnap.org/pmwiki/pmwiki.php) to obtain the boundaries of the lumen and inner and outer walls of the aorta. Four regions of interest were placed in the slice with maximum wall thickening and carefully outlined by one radiologist with 9 years of experience, encompassing different structures: the lumen, inner wall of the aorta, outer wall of the aorta, and peri-aortic fat. Quantitative parameters, including the mean, standard deviation (SD), signal-to-noise ratio (SNR), and contrast-to-noise noise ratio (CNR), were automatically calculated using Python code (version 3.6.4). The SNR of the vessel wall, CNR between the wall and lumen (CNR_inner_), and CNR between the wall and peri-aortic fat (CNR_outer_) were calculated as follows:

$${{{{\mathrm{SNR}}}}}=\frac{{{{{{\mathrm{Mean}}}}}}_{{{{{\mathrm{wall}}}}}}}{{{{{{\mathrm{SD}}}}}}_{{{{{\mathrm{wall}}}}}}}$$

$${{{{{\mathrm{CNR}}}}}}_{{{{{\mathrm{inner}}}}}}=\frac{{{{{|}}}{{{{\mathrm{Mean}}}}}}_{{{{{\mathrm{wall}}}}}}-{{{{{\mathrm{Mean}}}}}}_{{{{{\mathrm{lumen}}}}}}{{{|}}}}{\sqrt{\frac{1}{2}({{{{{\mathrm{SD}}}}}}_{{{{{\mathrm{wall}}}}}}^{2}+{{{{{\mathrm{SD}}}}}}_{{{{{\mathrm{lumen}}}}}}^{2})}}$$

$${{{{{\mathrm{CNR}}}}}}_{{{{{\mathrm{outer}}}}}}=\frac{{{{{|}}}{{{{\mathrm{Mean}}}}}}_{{{{{\mathrm{wall}}}}}}-{{{{{\mathrm{Mean}}}}}}_{{{{{\mathrm{fat}}}}}}{{{|}}}}{\sqrt{\frac{1}{2}({{{{{\mathrm{SD}}}}}}_{{{{{\mathrm{wall}}}}}}^{2}+{{{{{\mathrm{SD}}}}}}_{{{{{\mathrm{fat}}}}}}^{2})}}$$

### Radiation dose

The CT dose index (CTDI_vol_) and dose-length product (DLP) were obtained for each patient. The effective radiation dose (ED) was calculated as the DLP multiplied by a conversion factor of 0.015 (mSv · mGy^−1^ · cm^−1^) [23].

### Statistical analysis

Statistical analyses were performed using R software (version 3.6.1; http://www.R-project.org). Quantitative data are expressed as the mean ± SD or median and interquartile range, when appropriate. The Shapiro–Wilk test was used to assess the normal distribution of the data. For continuous variables with a normal distribution, one-way ANOVA was used, and pairwise *t*-tests with Bonferroni correction were used for multiple comparisons. The Friedman test was applied to non-normally distributed data, and the Wilcoxon signed-rank test with Bonferroni correction was used for multiple comparisons. Pairwise comparisons of HIR and DLR were used to illustrate the impact of the reconstruction algorithms, whereas pairwise comparisons between DB and other phases were performed to demonstrate the value of the DB technique. Statistical significance was set at *p* < 0.05.

Inter-observer agreement of qualitative image analyses was assessed by kappa coefficients, and that of vessel wall thickness measurement was calculated by the intraclass correlation coefficient (ICC) (criteria: ≤ 0.40, poor; 0.41–0.60, moderate; 0.61–0.80, good; > 0.80, excellent).

## Results

### Baseline characteristics and radiation dose

A total of 50 patients diagnosed as TAK (*n* = 48) and GCA (*n* = 2) were finally enrolled, 86% were females with a mean age of 33.5 ± 11.4 (range 18–60) years.

**Fig. 1:**
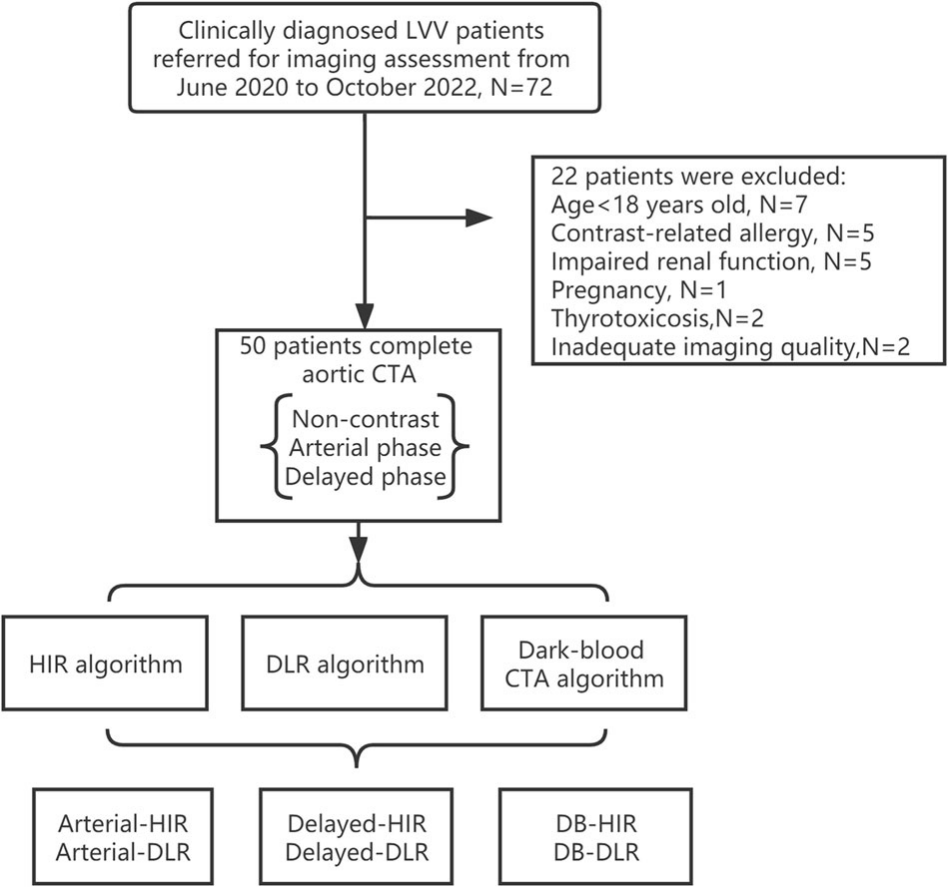
Flowchart of this study

The mean BMI was 19.6 ± 4.8. The CTDI_vol_ was 7.79 ± 1.43 mGy, DLP was 603.92 ± 118.52 mGy · cm, and ED was 9.06 ± 1.78 mSv. Figure 1 shows the patient inclusion flowchart.

### Qualitative image quality analysis

#### Comparison between HIR and DLR

For all image quality characteristics, including outer-wall delineation, inner-wall delineation, and overall image quality, the DLR scores were significantly higher than those of the HIR for all three phases (all *p* < 0.05).

#### Comparison between DB phase and conventional CTA phases

Regarding the outer wall delineation, there were no significant differences between the DB scores and the other two phases (all *p* > 0.05). For inner-wall delineation, the general trend was that the arterial phases ranked first, followed by the DB, and the last one was the delayed phases. For overall image quality, DB scores were significantly higher than delayed-phase scores (all *p* < 0.001); however, they exhibited no significant difference from arterial phase scores (all *p* > 0.05; except DB-DLR vs. A-DLR, reader 2, *p* = 0.02).

#### Inter-observer agreement

The inter-observer agreement for qualitative image quality was good to excellent for outer-wall delineation (kappa = 0.80), inner-wall delineation (kappa = 0.80), and overall image quality (kappa = 0.83).

**Fig. 2:**
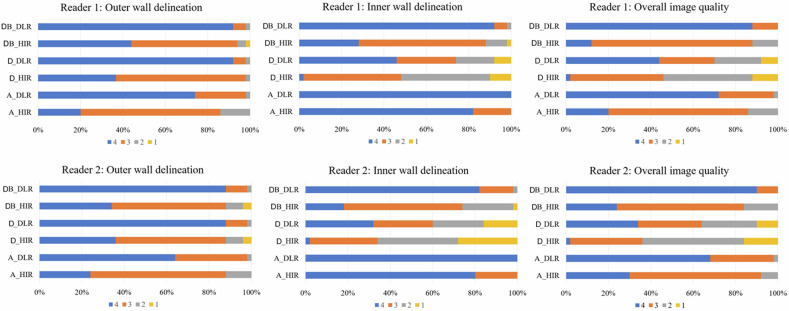
Stacked bar graph shows qualitative image quality scores (4-point scale)

**Fig. 3:**
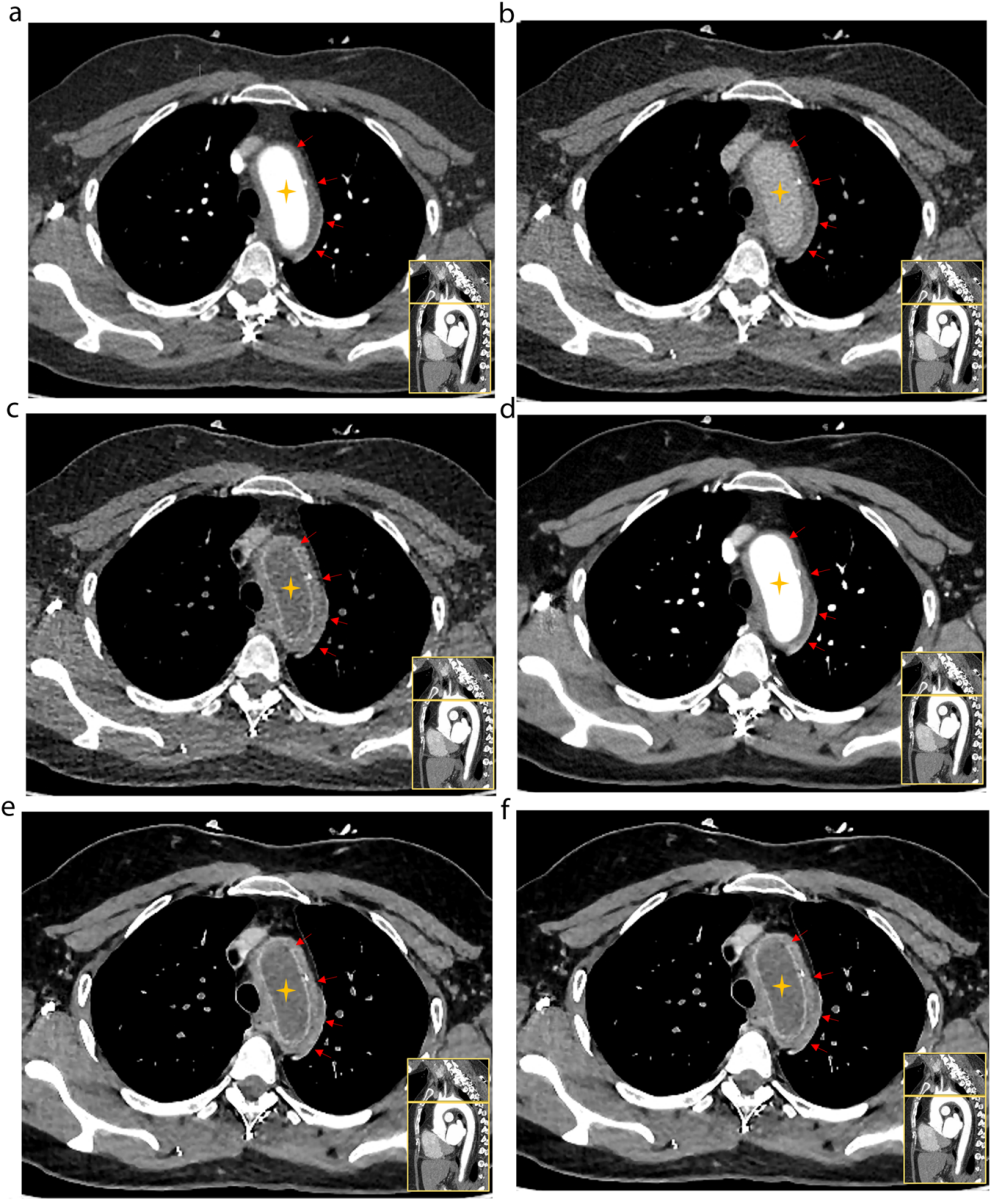
Images of a 36-year-old woman, diagnosed with Takayasu’s arteritis (TAK) showing a slice of the aortic arch with maximal wall thickening. **a** Arterial phase image reconstructed with HIR (A-HIR), (**b**) delayed-phase image reconstructed with HIR (D-HIR), (**c**) dark blood image reconstructed with HIR (DB-HIR), (**d**) arterial phase image reconstructed with DLR (A-DLR), (**e**) delayed-phase image reconstructed with DLR (D-DLR), and (**f**) dark blood image reconstructed with DLR (DB-DLR). Horizontally, the comparison between dark blood images and conventional arterial- and delayed-phase images, vascular wall enhancement, and the details of the obvious enhancement of the intima arteriae are depicted more clearly in the dark blood images (**c**, **f**) than in the traditional arterial (**a**, **d**) and delayed images (**b**, **e**). Longitudinally, comparing the DLR (**a**, **b**, **c**) with HLR (**d**, **e**, **f**), DLR demonstrated a high signal-to-noise ratio, and the overall image quality. HIR, hybrid iterative reconstruction; DLR, deep-learning reconstruction

Detailed results of the qualitative image quality analysis are presented in Table 1 and Fig. 2. Figure 3 show representative images of the patient’s images.

**Table 1:** Qualitative image quality comparison

Measure	Arterial phase		Delayed phase		Dark blood		*p*	*p* (HIR vs. DLR)			*p* (arterial vs. DB)		*p* (delayed vs. DB)	
	A-HIR	A-DLR	D-HIR	D-DLR	DB-HIR	DB-DLR		A-HIR vs. A-DLR	D-HIR vs. D-DLR	DB-HIR vs. DB-DLR	A-HIR vs. DB-HIR	A-DLR vs. DB-DLR	D-HIR vs. DB-HIR	D-DLR vs. DB-DLR
Outer-wall delineation														
Reader 1	3.06 (0.59)	3.72 (0.50)	3.30 (0.61)	3.90 (0.36)	3.36 (0.66)	3.90 (0.36)	< 0.001	< 0.001	< 0.001	< 0.001	0.14	> 0.99	> 0.99	> 0.99
Reader 2	3.12 (0.59)	3.62 (0.53)	3.20 (0.76)	3.86 (0.40)	3.18 (0.75)	3.86 (0.40)	< 0.001	< 0.001	< 0.001	< 0.001	> 0.99	0.14	> 0.99	> 0.99
Inner-wall delineation														
Reader 1	3.82 (0.39)	4.00 (0.00)	2.40 (0.70)	3.12 (0.98)	3.14 (0.67)	3.90 (0.36)	< 0.001	0.02	< 0.001	< 0.001	< 0.001	0.62	< 0.001	< 0.001
Reader 2	3.80 (0.40)	4.00 (0.00)	2.08 (0.83)	2.76 (1.08)	2.90 (0.71)	3.80 (0.40)	< 0.001	0.01	< 0.001	< 0.001	< 0.001	0.03	< 0.001	< 0.001
Overall image quality														
Reader 1	3.06 (0.59)	3.70 (0.51)	2.36 (0.72)	3.06 (1.00)	3.00 (0.49)	3.88 (0.33)	< 0.001	< 0.001	< 0.001	< 0.001	> 0.99	0.22	< 0.001	< 0.001
Reader 2	3.22 (0.58)	3.66 (0.52)	2.22 (0.74)	2.88 (1.00)	3.08 (0.63)	3.90 (0.30)	< 0.001	< 0.001	< 0.001	< 0.001	> 0.99	0.02	< 0.001	< 0.001

*HIR* hybrid iterative reconstruction, *DLR* deep-learning reconstruction, *A* arterial phase, *D* delayed phase, *DB* dark blood

### Quantitative image analysis

#### Comparison between HIR and DLR

As shown in Table 2, compared to HIR, DLR significantly increased CT values, decreased image noise, increased SNR (except for A-HIR vs. A-DLR, *p* = 0.70), increased CNR_outer_, and increased CNR_inner_ in all three phases (all *p* < 0.001).

**Table 2:** Quantitative image quality comparison

Measure	Arterial phase		Delayed phase		Dark blood		*p*	*p* (HIR vs. DLR)			*p* (Arterial vs. DB)		*p* (Delayed vs. DB)	
	A-HIR	A-DLR	D-HIR	D-DLR	DB-HIR	DB-DLR		A-HIR vs. A-DLR	D-HIR vs. D-DLR	DB-HIR vs. DB-DLR	A-HIR vs. DB-HIR	A-DLR vs. DB-DLR	D-HIR vs. DB-HIR	D-DLR vs. DB-DLR
CT_vascular wall_	53.64 (20.90)	51.30 (20.01)	67.25 (17.85)	71.01 (18.76)	69.81 (29.36)	79.23 (19.98)	< 0.001	< 0.001	< 0.001	< 0.001	0.007	< 0.001	< 0.001	< 0.001
SD_vascular wall_	55.83 (19.78)	50.03 (18.23)	38.47 (9.28)	33.92 (9.53)	42.77 (11.07)	34.15 (11.22)	< 0.001	< 0.001	< 0.001	< 0.001	< 0.001	< 0.001	< 0.001	> 0.99
SNR_vascular wall_	1.09 (0.52)	1.18 (0.61)	1.80 (0.58)	2.20 (0.74)	1.73 (0.76)	2.45 (0.80)	< 0.001	0.70	< 0.001	< 0.001	< 0.001	< 0.001	> 0.99	< 0.001
CNR_outer_	3.02 (1.18)	3.34 (1.23)	3.93 (1.20)	4.90 (1.74)	3.72 (1.29)	5.25 (1.95)	< 0.001	< 0.001	< 0.001	< 0.001	< 0.001	< 0.001	0.01	0.006
CNR_inner_	7.79 (2.70)	9.44 (3.71)	1.79 (0.80)	2.09 (0.90)	2.20 (1.05)	3.42 (1.30)	< 0.001	< 0.001	< 0.001	< 0.001	< 0.001	< 0.001	0.11	< 0.001

*HIR* hybrid iterative reconstruction, *DLR* deep-learning reconstruction, *A* arterial phase, *D* delayed phase, *DB* dark blood, *CT*_*vascular wall*_ CT attenuation of vascular wall, *SD*_*vascular wall*_ standard deviation of vascular wall, *SD*_*vascular wall*_ signal-to noise ratio of vascular wall, *CNR*_*outer*_ signal-to-noise ratio of vascular wall

#### Comparison between DB and conventional CTA phases

Compared with the arterial phases, DB yielded significantly higher CT values, lower image noise, higher SNR, and higher CNR_outer_ (all *p* < 0.05). Nevertheless, the CNR_inner_ was much lower in the DB phase than in the arterial phase (both *p* < 0.001). When compared with the delayed phases, DB had significantly higher CT values (both *p* < 0.001), higher (DB-HIR vs. D-HIR, *p* < 0.001), comparable image noise (DB-DLR vs. D-DLR, *p* > 0.99), higher (DB-DLR vs. D-DLR, *p* < 0.001), comparable SNR (DB-HIR vs. D-HIR, *p* > 0.99), higher DLR-based CNR_outer_ (DB-DLR vs. D-DLR, *p* = 0.006) and CNR_inner_ (DB-DLR vs. D-DLR, *p* < 0.001).

### Vessel wall thickness

The maximum wall thickness of different image datasets ranged from 3.45 ± 2.10 mm to 4.83 ± 2.21 mm, for reader 1, and ranged from 3.79 ± 2.38 mm to 4.54 ± 2.34 mm for reader 2. The ICC values between the two readers were 0.85, 0.89, 0.89, 0.90, 0.88, and 0.92 for the six sequences (A-HIR, A-DLR, D-HIR, D-DLR, DB-HIR, and DB-DLR), respectively. The detailed results are presented in Table 3.

**Table 3:** Slice thickness comparison

Measure	Arterial phase		Delayed phase		Dark blood		*p*	*p* (HIR vs. DLR)			*p* (Arterial vs. DB)		*p* (Delayed vs. DB)	
	A-HIR	A-DLR	D-HIR	D-DLR	DB-HIR	DB-DLR		A-HIR vs. A-DLR	D-HIR vs. D-DLR	DB-HIR vs. DB-DLR	A-HIR vs. DB-HIR	A-DLR vs. DB-DLR	D-HIR vs. DB-HIR	D-DLR vs. DB-DLR
Reader 1	3.51 (2.05)	3.45 (2.10)	3.72 (2.14)	3.72 (2.35)	4.83 (2.47)	4.61 (2.21)	< 0.001	0.91	> 0.99	0.12	< 0.001	< 0.001	< 0.001	< 0.001
Reader 2	4.12 (2.11)	4.10 (2.20)	3.85 (2.31)	3.79 (2.38)	4.54 (2.34)	4.33 (2.15)	< 0.001	> 0.99	> 0.99	0.01	< 0.001	0.02	< 0.001	0.006
ICC	0.85 (0.75, 0.91)	0.89 (0.81, 0.94)	0.89 (0.81, 0.94)	0.90 (0.83, 0.94)	0.88 (0.80, 0.93)	0.92 (0.86, 0.95)	/	/	/	/	/	/	/	/

*HIR* hybrid iterative reconstruction, *DLR* deep-learning reconstruction, *A* arterial phase, *D* delayed phase, *DB* dark blood

## Discussion

This study demonstrated that the DB CTA combined with DLR depicts LVV by objectively evaluating vessel wall inflammation. The image quality of the DB CTA technique was superior to that of the arterial and delayed phases in most aspects, except for inner-wall delineation, for which the arterial phase had a specific advantage. Furthermore, the overall image quality was optimal for DB CTA images, and further improvements were achieved by combining DB with DLR reconstruction.

Our proposed DB CTA technique was based on a commercial post-processing application called CE-boost, which was previously used to improve the visualization of type II endoleaks [18] and abdominal CTA [24]. By using registration, subtraction, and reading operations, the aortic wall was further enhanced, and the CT attenuation of DB significantly increased compared to the arterial and delayed phases. Simultaneously, the signal in the aortic lumen was suppressed to mimic dark-blood MR images. Our method integrated the information of the arterial and delayed phases and was different from the dual-energy solution proposed by Rotzinger et al [16], who applied two-material decomposition to generate DB images. This dual-energy approach could improve the lumen-to-wall contrast compared with the non-contrast phase; however, only the arterial phase was utilized, which cannot truly reflect contrast medium transport in the vessel wall. Moreover, the DB technique used in this study is a software-only solution that can be easily integrated into a clinical workflow without requiring dedicated hardware.

Our study highlights the potential benefit of DB aortic CT in evaluating large-vessel wall thickening compared to conventional arterial and delayed-phase images. In the arterial phase, the contrast agent was mainly concentrated in the lumen, enhancement of the vessel wall itself was not noticeable, and the boundary between the outer edge of the vessel wall and surrounding fat was not displayed. In the delayed phase, the concentration of the contrast agent in the arterial lumen is reduced, and the vessel wall is enhanced, resulting in a more apparent boundary between the outer edge of the vessel wall and surrounding fat; however, the sharpness between the inner edge of the vessel wall and lumen decreases. The DB sequence can clearly depict both the outer and inner boundaries of the vessel wall by inhibiting the signal of the lumen to more clearly highlight the structure of the vessel wall and abnormalities, such as vessel wall thickening, arterial aneurysm, and lumen stenosis, thereby enhancing confidence in the imaging diagnosis of arteritis [16, 17].

Recent studies [25, 26] have shown that DLR improves the image quality of the aorta compared with HIR. Heinrich et al [26] demonstrated a decrease of 38–54% in noise and doubled the CNR for the aorta with DLR. A coronary CTA study by Xu et al [25] also reported that aortic imaging with DLR had lower noise and higher SNR and CNR than other reconstruction algorithms. In the present study, we focused on the aortic wall and not the lumen, as in previous studies, and found that with the DLR, SNR, and CNR of the outer wall, the CNR of the inner wall increased by 8%, 11%, and 21%, respectively, for the arterial phase. The rates were 22%, 25%, and 17% in the delayed phase, and 42%, 41%, and 55% in the DB phase, respectively. This finding indicated that DLR not only improved the visualization of the details of the aortic wall in the traditional arterial and delayed phases but also showed remarkable advantages in the newly proposed DB technique. The combination of DLR and DB would benefit from the need for subtle structural abnormalities of the aortic wall [16, 17, 26], and is recommended by our study for evaluating patients with LVV.

This study has several limitations. First, the single-center design resulted in limited sample size and omitted subanalyses for LVV subtypes and anatomical variations (abdominal aorta, thoracic aorta, and aortic arch). Second, we did not validate the vessel wall thickness using MRI because of the relatively longer registration time and higher cost of MRI in our institutional workflow. Future research will include a comparative study of DB CTA and MRI. Third, no personalized design for the contrast injection rate or delay time hindered the best image quality. Fourth, the performance of the DB technique and DLR for LVV under low-dose conditions requires further evaluation.

In conclusion, our findings align with our initial hypothesis that integrating the DB CTA technique with the DLR algorithm enhances the imaging quality in aortic CTA examinations, particularly for the depiction and measurement of vessel wall inflammation. The study confirmed that the DB CTA technique surpasses the arterial and delayed phases in terms of overall and outer-wall visualization. Although the clarity of the inner-wall delineation might be marginally less distinct when compared to the arterial phase, the combined application of DB imaging and DLR has proven to be the most effective, outperforming other imaging sequences in delivering superior image quality and vessel wall visualization for the evaluation of LVV.

## Data Availability

We are committed to the principles of transparency and reproducibility in scientific research. Therefore, we are pleased to inform you that all data and materials related to our study are available upon request. Interested researchers can obtain them by contacting the corresponding author, L.K., at klyan@163.com.

## Abbreviations

CE-boost: Contrast-enhancement-boost
CNR: Dark blood
CTA: Computed tomography angiography
DB: Dark blood
DLR: Deep-learning reconstruction
GCA: Giant cell arteritis
HIR: Hybrid iterative reconstruction
LVV: Large-vessel vasculitis
SNR: Signal-noise-ratio
TAK: Takayasu arteritis
